# AGING-RELATED ADIPOSE REMODELING AND DYSFUNCTION

**DOI:** 10.1093/geroni/igac059.1262

**Published:** 2022-12-20

**Authors:** Qiong (Annabel) Wang

## Abstract

Aging is associated with insulin resistance, cardiovascular dysfunction, and many other chronic metabolic disorders, significantly shortening healthspan and lifespan. Fat (adipose) tissue, as the major site for energy storage, maintains whole-body energy homeostasis and insulin sensitivity. Adipose tissue has extraordinary plasticity, and it was not until recently that fat tissue remodeling during aging is considered to play an essential role in aging-associated metabolic disorders. Benefiting from recent technology advances, especially the single-cell technology and comprehensive genetic mouse models, we are beginning to unmask how adipose tissue remodels during aging cellularly and molecularly. This symposium features internationally-renowned aging research scientists whose work focuses on how aging remodels adipose tissues and how adipose tissue is vital for healthy aging and longevity. We will hear from Philipp Scherer from The University of Texas Southwestern Medical Center, who will present his research on the impact of adipocyte-derived factors on Healthspan and Lifespan; Hei Sook SUL from the University of California Berkeley will discuss “Aging dependent changes in adipose precursors”; Annabel Wang from the City of Hope will introduce her recent discovery of a new type of adipocyte progenitor cell that promotes aging-related visceral adiposity; and lastly, Gina Wade from the University of Wisconsin-Madison who will talk about “Regulation of aging energy expenditure by plasma lipid signaling”. Attendees will learn about the latest breakthroughs in adipose tissue aging, and the role of adipose tissue in maintaining and restoring metabolic health in aged individuals.

